# Current Challenges to Align Inflammatory Key Events in Animals and Lung Cell Models *In Vitro*

**DOI:** 10.1021/acs.chemrestox.4c00113

**Published:** 2024-08-08

**Authors:** Isidora Loncarevic, Seyran Mutlu, Martina Dzepic, Sandeep Keshavan, Alke Petri-Fink, Fabian Blank, Barbara Rothen-Rutishauser

**Affiliations:** † Adolphe Merkle Institute, University of Fribourg, Chemin des Verdiers 4, 1700 Fribourg, Switzerland; ‡ Lung Precision Medicine (LPM), Department for BioMedical Research (DBMR), University of Bern, Bern, Switzerland; § Department for Pulmonary Medicine, Allergology and Clinical Immunology, Inselspital, Bern University Hospital, University of Bern, Bern, Switzerland; ∥ Chemistry Department, University of Fribourg, Chemin du Musée 9, 1700 Fribourg, Switzerland

## Abstract

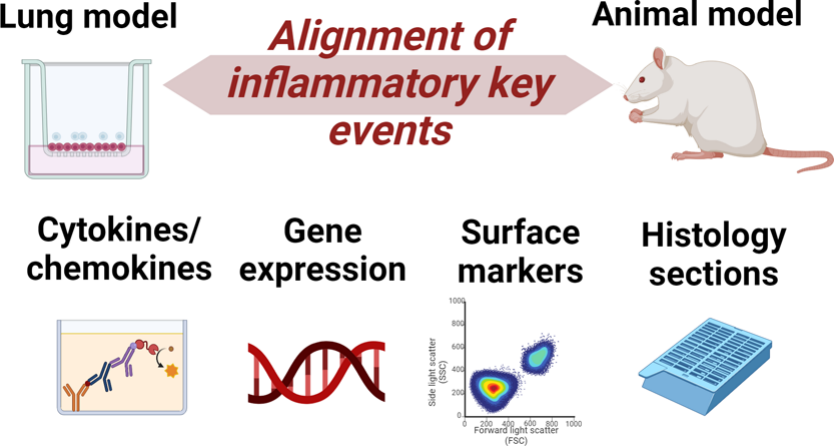

With numerous novel and innovative *in vitro* models emerging every year to reduce or replace animal testing, there is an urgent need to align the design, harmonization, and validation of such systems using *in vitro-in vivo* extrapolation (IVIVE) approaches. In particular, in inhalation toxicology, there is a lack of predictive and prevalidated *in vitro* lung models that can be considered a valid alternative for animal testing. The predictive power of such models can be enhanced by applying the Adverse Outcome Pathways (AOP) framework, which casually links key events (KE) relevant to IVIVE. However, one of the difficulties identified is that the endpoint analysis and readouts of specific assays in *in vitro* and animal models for specific toxicants are currently not harmonized, making the alignment challenging. We summarize the current state of the art in endpoint analysis in the two systems, focusing on inflammatory-induced effects and providing guidance for future research directions to improve the alignment.

## 1 Introduction

During the past years, the approach of regulatory toxicology for hazard and risk assessment of, e.g., chemicals, biocides, materials, or pharmaceuticals, has been mainly based on animal testing.^1^ Acute inhalation toxicity testing for regulatory purposes is performed in line with OECD (Organisation for Economic Co-operation and Development) test guidelines (TGs), e.g., TG433.^2^ There is increasing production and application of new substances, e.g. chemicals, pesticides, or materials, and given their wide variety, the resources required for traditional safety assessments (i.e., animal testing) will increase significantly as the field of inhalation toxicology is facing challenges regarding the design of reliable *in vitro* test systems.^3^ Intensified efforts have been made toward systematic development and evaluation of relevant and more reliable nonanimal models that have progressed impressively over the last 20 years, and the variety of invertebrate animal models,^4^*in silico*,^5^ and *in vitro* human-based methods are enormous.^6^

For these alternative approaches to be more broadly used and accepted by the academic, industrial sector and regulatory bodies, orchestrated efforts are required to show the robustness and reliability of *in vitro* methods. The Good *In vitro* Method Practices (GIVIMP) guidance document supports test developers in this direction.^7^ Another way to increase the use of alternatives is to coordinate validation studies and gain regulatory approval and installment as TGs or standard methods.^8−10^ The number of (pre)validated alternative methods to partially or fully replace animal testing has increased in recent years.^11^ Most approved methods were developed to support the revised skin and eye irritation TGs.^12^ Still, other predictive and (pre)validated tests, e.g., for intestine or lung, are lagging, although using (human) epithelial tissue models for safety assessments has found valid applications.^13^

One important aspect for a broad acceptance of *in vitro* lung models is the demonstration of predictivity, i.e., the outcome in an *in vitro* model must reflect the effect *in vivo*, which can be in humans or animals. In this perspective, we describe which *in vitro* and *in vivo* assays can currently be performed to assess inflammatory-induced effects with the aim of proposing a suitable approach for aligning endpoints between the two systems. The limitations of the assays are summarized, and recommendations for improving future alignment are given.

## 2 The Concept of *In Vitro*-*In Vivo* Extrapolation

The goal of predicting the observed *in vitro* effects of inhaled toxicants on the whole organism has led to improved concepts of *in vitro*-*in vivo* extrapolation (IVIVE), which have contributed toward a significant reduction of the use of laboratory animals^14^ in the fields of pharmaco-dynamics and -kinetics assessment^15^ and in hazard and risk research.^16^ Recently, Ma-Hock and colleagues proposed a six-step IVIVE procedure:^17^

1) Determine *in vivo* exposure;
2) Identify *in vivo* organ burden at the lowest observed adverse effect concentration;
3) Extrapolate *in vivo* organ burden to *in vitro* effective dose;
4) Extrapolate *in vitro* effective dose to nominal concentration;
5) Set dose ranges to establish dose–response relationships;
6) Consider uncertainties and specificities of the *in vitro* test system.

As IVIVE can align the differences in *in vivo* and *in vitro* exposures, it serves the 3Rs principle to replace and reduce animal testing^18^ as implemented in the European Union Directive 63/2010/EU on the protection of animals used for scientific purposes.

Also, human relevance is a most important criterion for regulatory acceptance, and recently, the OECD launched a program to describe Adverse Outcome Pathways (AOPs).^19^ This framework takes human epidemiology and *in vivo* animal data into consideration to describe causally connected key events (KEs) resulting in a specific adverse outcome (AO).^20,21^ Such a toxicological response is initiated with a biological event at the molecular level after exposure to stressors.^22^

AOPs are versatile, modular, and evolving documents that can be continuously updated with new data.^23,24^ AOPs can form the basis of toxicity screening for substances to be prioritized for animal testing and guide the principles of decision matrices such as Integrated Approaches to Testing and Assessment (IATA).^25−27^ In addition, AOPs can indicate which specific key event or even molecular initiating event (MIE) is detected by a certain readout of an *in vitro* system,^23,28^ thus supporting the validity of an alternative test method. The selection of AOP-relevant *in vitro* assays has also recently been described with a focus on engineered nanomaterials.^29^ This review describes the importance of relevant *in vitro* assays describing a certain adverse outcome and how gained information can be used for IVIVE.

**Figure 1:**
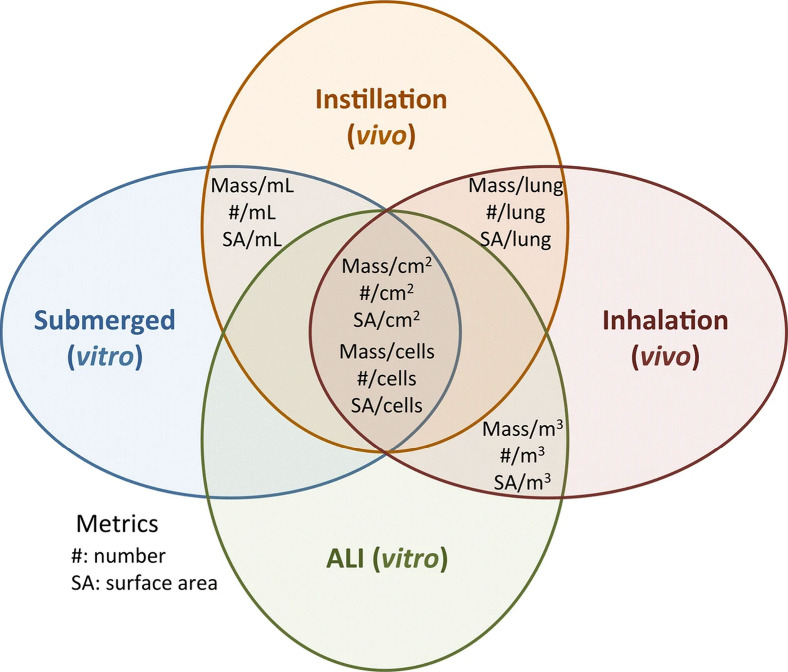
Relevant dose metrics for IVIVE. From Loret et al.^31^ under the terms of the Creative Commons Attribution 4.0 International License (http://creativecommons.org/licenses/by/4.0/).

One of the challenges of IVIVE is the compatibility of different dose metrics between *in vivo* and *in vitro* approaches. It is important to use common dose metrics for the most efficient comparison of *in vitro* and *in vivo* conditions. Dose metrics often include concentrations, expressed as mass/volume of liquid for *in vitro* in submerged conditions and mass/volume of air for *in vivo* inhalation studies. These metrics cannot be used within the different *in vivo* (inhalation vs instillation) and *in vitro* (ALI vs submerged) methodologies. Also, the use of mass/volume concentrations is problematic, as it does not consider the actual contact and interaction between the inhaled toxicants and the cells or tissues when grown under submerged conditions. This has been debated in depth for (nano)materials.^30^ Hence, using such dose metrics for IVIVE, particularly for poorly soluble inhaled toxicants, e.g., particles, is challenging since their toxicity depends on their surface reactivity. For *in vivo* studies, the total mass of inhaled particles administered per lung, animal, or kg body weight as a dose metric is employed. This approach considers whole organ deposition but cannot be applied *in vitro*. To employ common dose metrics in IVIVE, the total mass deposited on cells *in vitro* or the lung *in vivo* can be normalized to the surface area of the tissue in *vitro/in vivo* or to the number of cells in *vitro/in vivo*. In the case of particles, doses expressed in mass can also be normalized to the surface area of inhaled particles, which is the most effective dose metric for acute inhaled particle toxicity in the lung^31^ (Figure 1). A good example of efficient dose metrics has been demonstrated in a recent study. The Lowest Observed Adverse Effects levels (LOAELs) for titanium dioxide and cerium dioxide and dose intervals determined in *vitro* ALI cocultures of pulmonary epithelial cells and macrophages were compared with an *in vivo* inhalation model (rat). They were closer to *in vivo* when the doses were normalized to the number of macrophages present in the models (mg/10^6^ macrophages) than when normalized to the alveolar surface area.^31^ These findings demonstrate the importance of applying the optimal dose metrics for IVIVE.

## 3 Acute Inhalation Toxicology in Animals and *In Vitro* Lung Cell Models

**Figure 2:**
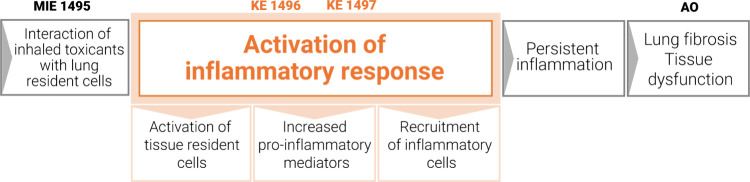
Inflammation-related KEs - Simplified scheme of AOP173 with the highlight of three major events in the inflammatory response. This scheme was adapted from Villeneuve et al., 2018^37^ published in open access that can be used under the terms of the Creative Commons Attribution 4.0 International License (http://creativecommons.org/licenses/by/4.0/).

To move away from animal testing, (new) alternative lung cell culture methods and the development of internationally accepted TGs currently require validation against data generated using animal models or derived from humans. For this purpose, the endpoint analysis from different systems must be aligned with the *in vitro* counterpart to improve IVIVE. However, due to significant differences in the *in vitro* and *in vivo* systems and handling of the samples collected after the toxicant exposure, the endpoint analysis might be very different and difficult to compare. To discuss these challenges, inflammation-related KEs (Figure 2) that have been reported in different AOPs, e.g., AOP 173 (https://aopwiki.org/aops/173), are discussed in this perspective. The KEs are linked to the observed acute and long-term adverse effects associated with inhaled toxicant exposure (from previous *in vivo* findings).^32−34^ They are relevant regarding the OECD TGs for inhalation toxicology. Some KEs were identified as important for IVIVE; however, *in vitro* approaches did not report on many KEs via specific biomarkers as most studies focus on a few cytokines/chemokines. The most relevant key events for inflammation after the MIE of an inflammatory stressor can be summarized as follows:^35,36^

- Tissue-resident cell activation
- Increased (pro)inflammatory mediators
- Leukocyte recruitment/activation

**Figure 3:**
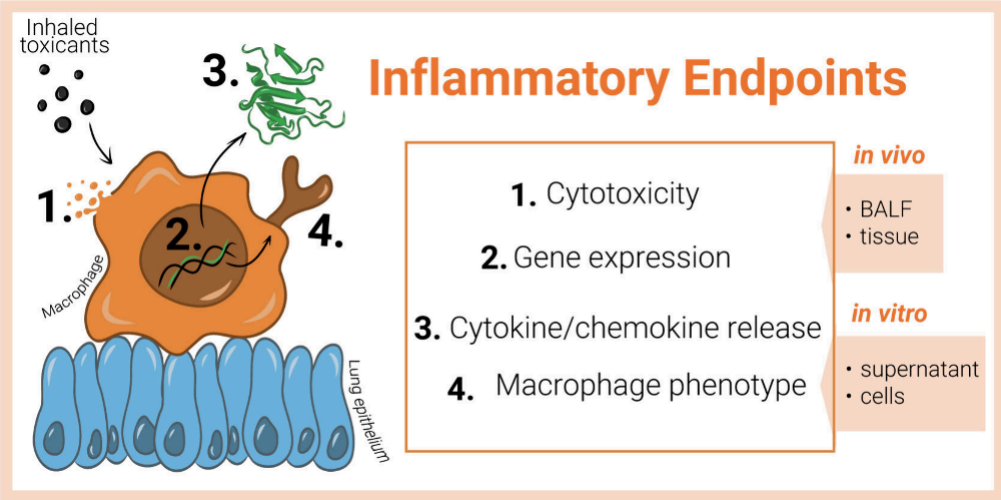
Key inflammatory endpoints identified by relevant assays to detect major changes in cell morphology, metabolism, and secretion. The assays to analyze cytotoxicity, cytokine and chemokine release, gene expression changes, and characterization of macrophage phenotype, can be measured in supernatant and collected cells from *in vitro* cell culture experiments to compare readouts with results acquired from bronchoalveolar lavage fluid (BALF) and tissue collected *in vivo*.

It is important to add that some of the KEs presented here may overlap or coincide. Selecting assays to measure the KEs in an AOP can help design testing strategies to predict complex outcomes such as inflammation. In the following subchapters, we present sets of assays following those KEs in animals and *in vitro* models (Figure 3) to discuss the challenges and recommendations for alignment.

## 4 Inflammation-Induced End Points in Animals

Currently, inhalation toxicity testing for regulatory purposes is performed in rats and/or mice, according to OECD TGs. One of the most widely used regulatory test protocols for inhaled toxicants is the *in vivo* subchronic toxicity 90-day inhalation study (OECD TG 413). In this latest guideline, 80 animals must be used to test an individual inhaled toxicant. It proposes a standard rat strain but allows alternatives if justified. Each group of 10 male and 10 female rats aged 7–9 weeks is exposed 6 h/day, 5 days/week for 13 weeks. Control animals receive filtered air.

### Cytotoxicity and Inflammatory Endpoints in Animals

In accordance with the OECD TG413, the evaluation of inflammatory endpoints in animals focuses on bronchoalveolar lavage fluid (BALF) analysis and histopathology. Lung tissue is systematically sampled, with the right lung lobes allocated for BALF collection and the left lobe for histological preparation (OECD, TG413).

#### Bronchoalveolar Lavage Fluid Analysis

The mandatory endpoints for BALF examination include (1) the lactate dehydrogenase test (LDH) to assess cytotoxicity and (2) total protein or albumin assay for assessing lung inflammation and injury caused by inhaled toxicant exposure. Increased LDH, protein levels, and albumin may be correlated with inflammatory processes postinhaled toxicant exposure. Another mandatory endpoint for BALF is (3) the total cell counts, including differential counts of macrophages, lymphocytes, eosinophils, and neutrophils, which may indicate an inflammatory response. A major problem with using commercially available LDH assays is their proprietary formulation, which makes optimization difficult due to the unknown composition and concentrations of the substances. Also, the addition of serum can induce variability in LDH assay readings, further diminishing and reducing reproducibility in *in vivo* settings compared to *in vitro* systems.^38^ For both total protein and albumin assays, the presence of other factors in the lung, such as mucus, contaminants, and substances associated with local inflammatory processes, can complicate the interpretation of the results.^39^

#### Histopathology

A thorough examination of nasopharyngeal, laryngeal, tracheal, and lung tissues is recommended for histopathological evaluation. Focus areas include immune cell infiltration, epithelial damage, and collagen deposition, indicating inflammation and fibrosis. Comprehensive examination protocols include multiple tissue levels to ensure thorough assessment, focusing on diverse epithelial cell types and draining lymphatic tissue. This approach is crucial as it allows for examining areas where immune cells circulate and may be directed to sites of injury caused by inhaled toxicants. In addition, different views of the trachea and regions within the left lung are examined (see TG413, Guideline 125). To highlight structures, tissue sections are stained with dyes such as hematoxylin and eosin (H&E), followed by histopathological examination to look for abnormalities such as cell infiltration, tissue thickening, or signs of inflammation.

**Figure 4:**
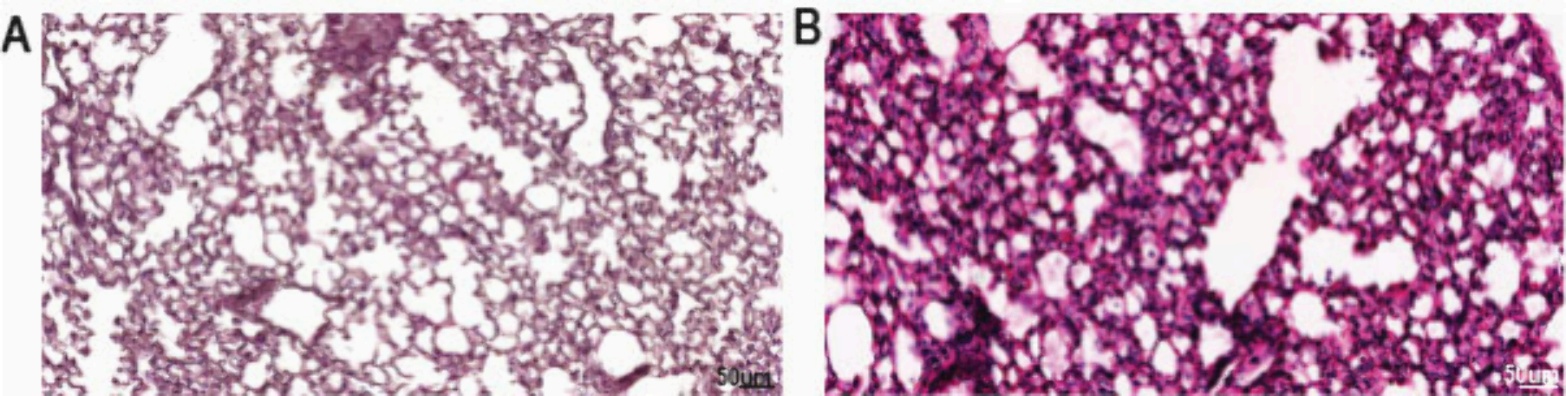
Hematoxylin and eosin (H&E) staining of healthy and bleomycin (BLM) treated mouse lungs. Mice were either instilled intratracheal with 50 μL of saline (control) or with BLM (1.52U/kg) to induce pulmonary fibrosis and sacrificed on day 14 following instillation. A. Control H&E-stained lung sections of mice treated as outlined above. B. H&E-stained lung sections of bleomycin mice.

Organs and tissues undergo histopathological evaluation in control and high-concentration groups, focusing on the respiratory tract, target organs, and gross lesions. If the animals in the high-exposure group are too severely affected by inhaled toxicants, the next lower concentration is analyzed to maintain the significance of the data. Lesions observed in high-concentration groups are examined across all groups. Figure 4 displays H&E staining for histopathological assessment of bleomycin-induced fibrosis in mice.

Although histopathology is an important and well-established diagnostic tool, it has several limitations. It is time-consuming and often requires extensive training. It is subject to variability depending on the sampling method, which can be affected by human subjectivity, and it can be expensive in terms of both money and time. In addition, without the integration of molecular diagnostics, histopathology alone may not provide the necessary reliability for comprehensive pathological assessment.^40^

Besides the previously mentioned mandatory endpoints proposed by OECD, BALF and sampled tissues could be used to perform additional analysis, such as cytokine secretion measured by enzyme-linked immunosorbent assay (ELISA), gene expression in tissue by quantitative Reverse Transcription Polymerase Chain Reaction (qRT-PCR), and immune cell characterization by flow cytometry. These additional experiments, which are not required by the OECD guidelines *per se*, provide valuable data, and we will discuss in detail how implementing them can support the IVIVE approach in the following paragraphs.

## 5 Inflammation-Induced Endpoints in *In Vitro* Lung Cell Models

The design of appropriate lung cell models is challenging as the interaction of inhaled toxicants with lung compartments depends on the physicochemical properties.^41^ The lung is a complex organ. Reproducing lung compartments *in vitro* requires a detailed understanding of its structure and composition. Many *in vitro* models of the human airway and lung parenchyma exist,^42,43^ and the opportunity to use these models not only for hazard assessment but also for preclinical research has been recognized.^44^ Such models range from simple mono- to more complex cocultures (healthy and diseased) based on primary lung cells or commercial cell lines representing the airway and alveolar region.^45^ In addition, companies offer fully reconstituted 3D human (small) airway tissues (MucilAir, e.g., MatTek Corporation, Epithelix Sàrl). Cocultures of epithelial with immune cells, i.e., macrophages and dendritic cells,^46^ mast cells,^47,48^ fibroblasts,^49^ or natural killer cells,^50^ have been described. In addition, lung models based on microfluidic devices,^51^ stimulating breathing mechanisms,^52,53^ and organoid cultures^54^ have become relevant due to their enhanced versatility. These advanced 3D lung cultures narrow the apparent gap between simple monocultures and animals.^55^ Despite all these enormous developments, none of the lung models have taken the first steps toward regulatory approval.^56^ One model, such as the alveolar coculture system composed of human macrophages, alveolar, and endothelial cells from the Gutleb lab to study respiratory sensitization,^57^ is, in our opinion, the most advanced one. In addition, the US Environmental Protection Agency (EPA) has recognized that the commercial MucilAir airway model can predict *in vivo* respiratory toxicity for the pesticide chlorothalonil and other contact irritants.^58^ Still, the predictivity of many toxicants has not yet been shown. In our laboratory, we have used a 3D human alveolar model (EpiAlveolarTM) made of primary cells to predict long-term responses to inhaled toxicants. The model was applied based on the AO concept for lung inflammation-induced fibrosis by applying repeated subchronic exposures to multiwalled carbon nanotubes (MWCNTs) and silica quartz particles (DQ_12_).^59^

Most lung cell models use permeable inserts to grow epithelial cells on their surface and to establish an air–liquid interface, i.e., cells on the upper surface are exposed to air, and cells are supplied with cell culture medium from the basal compartment. Usually, the supernatants, i.e., cell culture medium from the basal compartment and liquid from the apical side of the epithelial cells, are collected after an experiment. In addition, the cells can be fixed and prepared for microscopy investigations or be lysed for RNA or protein analysis.

In the text below, we made a short list of the most applied end-point analyses when using such cell models. Those endpoints are promising parameters for comparison to the *in vivo* endpoints as shown above (Figure 3)

### Cytotoxicity Endpoints

The cytotoxicity assessment serves as a crucial early indicator for understanding the impact of toxicants on the cellular compartment. These assays utilize colorimetric- or fluorescence-based detection methods, offering cost-effective and easy handling.^60^

LDH is an important enzyme of the anaerobic pathway. When the cellular plasma membrane is damaged, it is released into the cell culture medium. This release can be measured both *in vivo* from BALF and *in vitro* by assessing LDH in the supernatant of the cell culture.

Beyond cytotoxicity, evaluating the impact of toxicants on cell viability is an important endpoint. Among the numerous methods available, colorimetric viability assays such as MTT ((3-[4,5-dimethylthiazol-2-yl]-2,5 diphenyl tetrazolium bromide)^61^ and WST (water-soluble tetrazolium salts) are commonly used.^60^ Those approaches involve the cellular oxidoreductase and dehydrogenase enzymes in viable mammalian cells. Those enzymes catalyze the reduction of the water-soluble reagent to the product whose concentration can be determined through optical density measurement at the respective wavelength.

The advantage of these assays is that they are broadly used and are easy to implement in standard biological laboratories.^62^ When performing colorimetric or fluorescent-based methods for toxicant-exposed cells or tissues, it is crucial to test interference that can happen due to the toxicant’s intrinsic fluorescence/absorbance and interactions with assay components.^63^

### Inflammatory Endpoints

**Figure 5:**
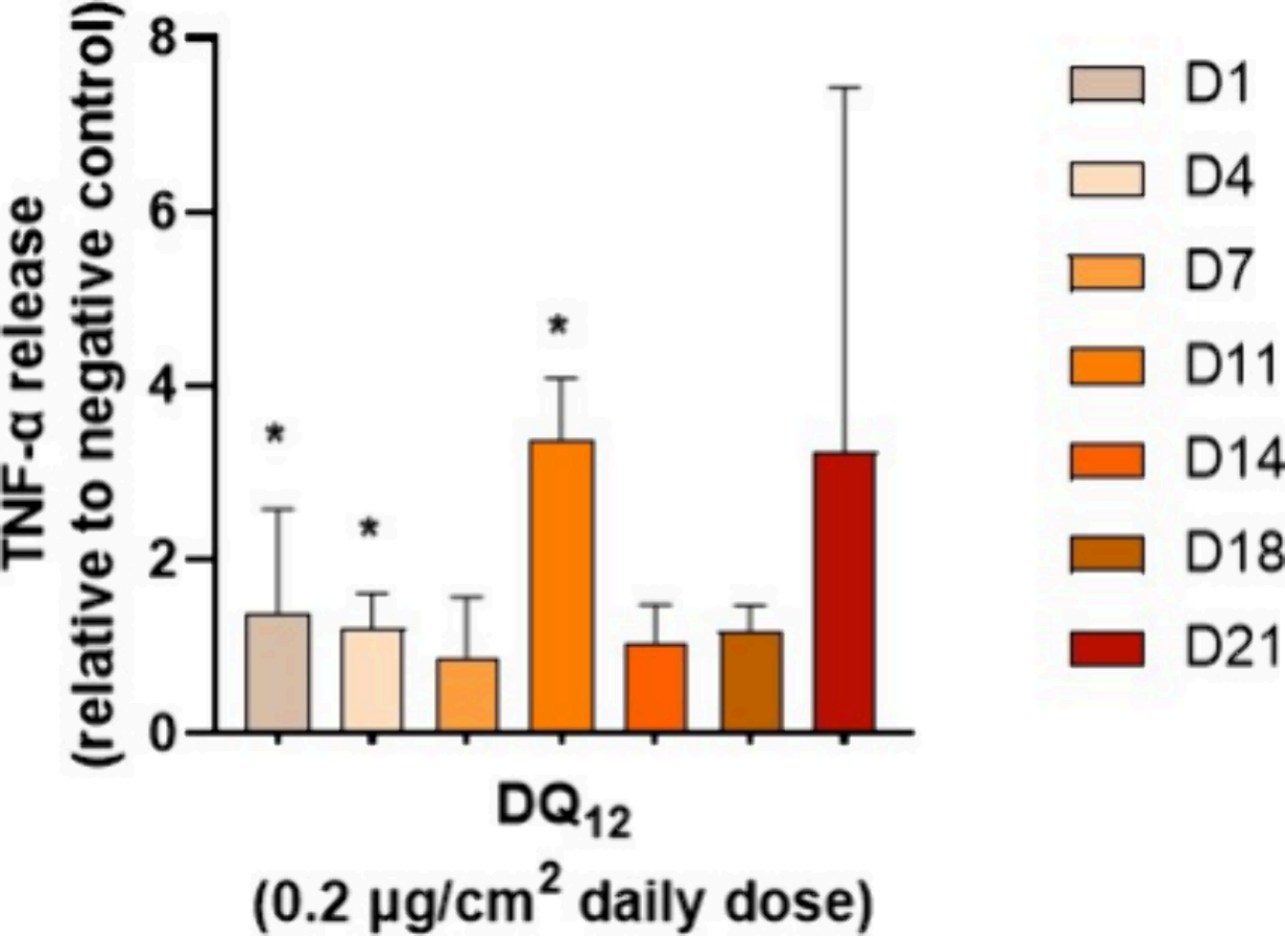
Tumor necrosis factor α (TNF- α) release was measured by ELISA. TNF-α released into the basal supernatants as a marker of proinflammatory response in an EpiAlveolar tissue was measured over 21 days (D1-D21) upon exposure to Dörentrup Quartz (DQ_12_) silica particles. Data are presented as relative to negative control. Data marked as with * were considered statistically significantly (*p* < 0.05) increased compared to negative control. Adapted with permission from ref (67), further permissions related to the material excerpted should be directed to the ACS.

For assessing inflammation, both the supernatant and cellular components can be used for the analysis. Various assays, such as Western blot analysis and multiplex protein array, can be employed, primarily focusing on the ELISA assay to measure cytokine and chemokine release (Figure 5). The ELISA assay is a standard method for evaluating cytokine/chemokine release onto the apical surface of the cells in case the cells are cultured at ALI or into the cell culture medium in the basal compartment. This technique is commonly used in many laboratories. The need for increased sensitivity and simplicity of the classic ELISA assay resulted in the development of novel methods, such as nanomaterial-enhanced ELISA^64,65^ and multiplex cytokine analysis. However, the use of methods with different sensitivities and different kits can make the comparability of the results across laboratories difficult;^66^ therefore, choosing the kits described in the literature is essential to provide reproducible data.

In addition to cytokine or chemokine release, changes in the gene expression of specific cytokines/chemokines are examined. RNA extraction is carried out from cell lysate. Quantification of gene expression is achieved through quantitative qRT-PCR. Known sequences of genes (e.g., IL-6, IL-8 for inflammation) are targeted for precise measurement.

With the development of *in vitro* models and their ability to be used for multiple weeks, it is possible to measure several time points beyond the traditional 4- or 24-h exposure.^29^ The duration for which *in vitro* models can be maintained varies significantly based on the specific system and the conditions under which it is cultured.^68^ Primary lung cell cultures can be kept stable at ALI for several weeks, as shown for the human airway epithelium derived from primary bronchial cells^69^ or for a recently established EpiAlveolar model, a 3D reconstructed model of human alveolar tissue consisting of alveolar epithelial cells, fibroblasts, and endothelial cells.^70^

It is also reported that some 3D lung spheroid and organoid models can be cultured even up to several weeks to a few months.^71^ Longer exposure times are particularly important since cytokine release and phenotypes of macrophages (see section c) change over time. However, defining specific exposure durations remains challenging, as the optimal duration of the experiment varies significantly with each tested toxicant and depending on the *in vitro* model used. Depending on their physical-chemical properties, different toxicants require different exposure times to elicit an inflammatory response. The type and number of cells used in each *in vitro* model can also differ between laboratories. Therefore, although longer exposure times of a minimum of a few days are preferable, this must be optimized for each experiment.

### Characterization of Inflammatory Cells

The previously mentioned *in vitro* lung cell models often add immune cells like macrophages, whether primary alveolar macrophages, monocyte-derived macrophages (MDMs),^72^ or human cell lines such as THP-1.^57^ Macrophages can be isolated from more complex cocultures, as described.^73^ The characterization of macrophages often relies on evaluating surface markers and intracellular proteins using flow cytometry. These markers help identify macrophages by phenotype and activation status. Additionally, intracellular markers are crucial in distinguishing between different macrophage phenotypes, such as M1 and M2, respectively.^74^ Also, functional aspects such as the phagocytic index are important parameters to assess general “fitness” and activation of free immune cells.^75^ For analysis of the expression profile of markers or phagocytic activity, flow cytometry enables precise identification and quantification of macrophage subsets. This capability facilitates a more profound comprehension of their functional roles, as seen in applications like studying their involvement in inflammation.

One limitation of this characterization of immune cells between two different systems would be that in the rat *in vivo* and the human *in vitro* macrophages do not express the same markers and still be quite similar in their functions. Rodents, even being close to the human, are still quite different, and therefore show different subsets of immune cells compared to cells of human origin. Also, the complex microenvironment in the rodents may shape the phenotype of macrophages differently compared to the simple *in vitro* microenvironment. These circumstances can make characterization and alignment difficult.

## 6 Additional Recommendations for Future Alignment Studies

Since *in vivo* and *in vitro* systems involve distinct methodologies and techniques, including animal handling, maintenance of specific sterile conditions, and employment of specific assay systems (e.g., BALF analysis for *in vivo* assessment), there is a need to adapt or expand the current experimental outline that is routinely done. While both *in vitro* and *in vivo* studies target similar outcomes (endpoints), their differing methodologies can result in variations in the observed responses. Therefore, a more comprehensive range of assays should be incorporated in future studies. In addition, transparency and collaboration between scientists and institutions working on similar projects *in vitro* and *in vivo* are of utmost importance. Using the same starting toxicant and measuring the same endpoints (e.g., cytokine secretion from BALF *in vivo* and cell culture supernatant *in vitro*) can be an optimal basis for ensuring comparability of the readouts between the two systems.

We have identified the following assays and readouts as promising approaches for harmonizing endpoint assessment with a focus on inflammatory KEs and have also summarized this in (Table 1):

**Table 1:** A Summary Table Describes the MIE and Early KEs Used to Assess Inflammation-Induced Effects in Animal and Cell Models

MIE and KEs	Animal models	Lung cell models
**MIE:** Interaction with the lung cell membrane	• Histological sections	• Histological sections
	• Immunofluorescence	• Immunofluorescence
	• TEM/SEM	• TEM/SEM
**KE1496:** Increased proinflammatory mediators	• BALF analysis (ELISA)	• Supernatant analysis (ELISA)
	• Gene analysis tissue (qRT-PCR)	• Gene analysis cells (qRT-PCR)
**KE1497:** Recruitment of inflammatory cells	• BALF analysis (Differential Counting)	• Macrophage characterization (Flow Cytometry)
	• Macrophage characterization (Flow Cytometry)	• Endothelial and epithelial cell characterization (Flow Cytometry)
	• Endothelial and epithelial cell characterization (Flow Cytometry)	

### Inflammatory Cytokines/Chemokines

Measuring cytokine/chemokine release using ELISA presents a versatile method applicable to both *in vitro* and *in vivo* approaches. For instance, it can be utilized on cell culture supernatants *in vitro* and BALF *in vivo*. Previous studies have highlighted promising correlations between IL-6 and IL-1β cytokine secretion observed in simple submerged macrophage models and *in vivo* data, particularly as it may correlate with the recruitment of inflammatory cells in the lungs.^76^ It is important to note that a cytokine/chemokine does not directly indicate inflammatory cell recruitment, but the observed correlations suggest a potential link between these factors. Also, one must interpret a resulting cytokine profile always in the light of the specific *in vitro* model employed, which may often lack key player immune cells, such as macrophages, dendritic cells, and T cells, and thereby have limitations, which preclude a classical (i.e., more complete) interpretation of a cytokine response as it is possible *in vivo*. For example, predicting Th1 and Th2 responses with an *in vitro* model will be challenging, as the *in vitro* setup would require the presence of many different immune cell types, as mentioned above, within a validated model. Hence it is crucial to carefully link the cytokine profile being assessed with the immune cells in the *in vitro* culture. The quality of the prevalidation and the resulting level of predictivity of an *in vitro* model regarding cytokine response will depend on its analysis and characterization upon exposure to as many different types of compounds as possible.

One limiting factor for this approach is interspecies difference. For example, IL-8, a crucial cytokine for immune recruitment in humans, is absent in rodents. Instead, rodents have different analogs that collectively mimic the main functions of human IL-8.^77^ Similar discrepancies exist for other important cytokines, making it difficult to fully align endpoints between human and rodent systems.

### Inflammatory Gene Expression

In addition to ELISA assays, examining changes in gene expression of specific cytokines/chemokines offers valuable insights and can be conducted in both *in vitro* and *in vivo* systems. This involves analyzing RNA extracted from cells in culture, from animal tissues, or immune cells obtained from BALF in animals. This approach allows researchers to assess the transcriptional regulation of cytokines and chemokines, providing a deeper understanding of the underlying mechanisms involved in inflammatory responses and disease progression.

While *in vitro* models excel at detecting subtle changes in gene expression, these changes might not translate to real-world toxicity due to the controlled and limited environment in an *in vitro* model. This highlights the importance of defining biological relevance for gene expression data, particularly in the field of inhalation toxicology.

To bridge this gap, we emphasize establishing thresholds beyond just statistical significance. Recent advancements advocate for applying p-value and fold-change criteria^78^ to ensure observed changes have a meaningful impact on pathways leading to disease (pathogenesis). The p-value indicates statistical significance, assessing the likelihood of observed changes being due to chance. Fold-change measures the magnitude of gene or protein expression changes, revealing their increase or decrease. Together, these criteria pinpoint statistically significant and biologically relevant changes to disease development.^79^ This combined approach with a focus on biological relevance strengthens the link between *in vitro* studies and real-world outcomes, ultimately leading to more robust data for inhalation toxicology research.

### Characterization of Inflammatory Cells

Recruitment of inflammatory cells is easily assessed *in vivo* through differential immune cell enumeration in BALF. However, aligning this readout with *in vitro* models is challenging for IVIVE as it is difficult to mimic the simultaneous recruitment of multiple immune cells *in vitro*. However, immune-system-on-chips are being designed and could solve this issue in the coming years.^80^ Focusing solely on one relevant cell type at a time, such as macrophages, while characterizing phenotype and functionality may facilitate *in vivo* and *in vitro* alignment. Existing literature categorizes macrophages into M1 and M2 activation states, offering a simplified approach.^81^ Flow cytometry, which analyses surface and intracellular markers, identifies and classifies macrophages. Common markers like CD11b, F4/80, CD68, and CD206 distinguish macrophages regarding their phenotypes, while intracellular markers like iNOS and Arginase-1 differentiate M1 and M2 phenotypes. This technique provides precise quantification of macrophage subsets, aiding in understanding their roles, and can be performed *in vivo* and *in vitro*. Even if the rats and human macrophages harbor the same markers, it does not mean they have the same function and thus refer to the same kind of macrophages. The markers employed for aligning both systems should be carefully chosen to remove the bias of the intrinsic functions of those immune cells between rats and humans.^82^

### Characterization of Endothelial Cells

Like macrophages, endothelial cells are a relevant cell type for measuring inflammatory responses as they express several highly relevant cell adhesion molecules (CAMs). Endothelial can be cultured at the basal side of permeable cell culture inserts.^83^ E-selectin, intercellular adhesion molecule 1 (ICAM-1), and vascular cell adhesion protein 1 (VCAM-1) are expressed on endothelial cells upon stimulation with inflammatory cytokines (e.g., TNFα, IL-1), and they play an important role in the adhesion of immune cells to the vascular endothelium. Their increased expression can be investigated by flow cytometry,^84^ qRT-PCR, or, circulating soluble forms of CAMs (sICAM and sVCAM), which can be measured by ELISA assay.^85^ Therefore, investigating molecules expressed on or released by endothelial cells that modulate immune cells can provide additional insights and help in the *in vivo* and *in vitro* alignment process.

### Histology

Histology may provide relevant information for the alignment of *in vitro* with *in vivo* results as new lung cell models can reliably mimic some of the tissue responses of a complex organism in a simplified and controlled environment. Even though it is not a routine method, *in vitro*, histology analysis can also be done with lung cell cultures. It can mainly be used to assess the spatial arrangement of the cells and the overall morphology of the model.^67^ Alignment of relevant endpoint analyses between an *in vitro* and an *in vivo* model, such as epithelial morphology or cell degradation, can help to improve predictivity *in vitro*. For instance, important indicators like tissue integrity, inflammatory markers, and cell structure can be investigated in both scenarios. The alignment of these indicators contributes to the predictivity and relevance of the *in vitro* model.

## 7 Conclusion

Achieving IVIVE poses a complex challenge, as it involves challenging alignment approaches between human *in vitro* lung cells and animal models at different levels. The choice of endpoints, readouts, and alignment methods may vary significantly depending on the specific model being used and characterized, the regulatory requirements, and the nature of the adverse outcomes under investigation. It is imperative to carefully plan experiments from both perspectives and adhere to standardized protocols wherever possible. Furthermore, rigorous prevalidation of findings is essential to ensure the reliability, reproducibility, and predictivity of results. Another approach to help us bridge the gap between *in vitro* and *in vivo* endpoints may be to use machine learning along the IVIVE process.^86^ Machine learning could enhance the IVIVE process, improving our ability to predict biological responses across different experimental settings. This advancement in data analysis bridges the gap between laboratory studies (*in vitro*) and real-life conditions (*in vivo*), facilitating more accurate drug discovery, toxicity assessment, and personalized medicine.^87,88^ Here, our approach has identified promising endpoints for biomarkers relevant to IVIVE. However, more research is required to prevalidate relevant assays and the *in vitro* models employed.

## Abbreviations

ALI: air–liquid-interface
AO: adverse outcome
AOP: adverse outcome pathway
BALF: bronchoalveolar lavage fluid
BLM: bleomycin
CAMs: cell adhesion molecules
ELISA: enzyme-like immunosorbent assay
ENM: engineered nanomaterial
EPA: US Environmental Protection Agency
GIVIMP: Guidance Document on Good *In Vitro* Method Practices
H&E: hematoxylin & eosin
IATA: integrated approaches to testing and assessment
IVIVE: *in vivo* to *in vitro* extrapolation
KE: key event
LDH: lactate dehydrogenase test
MDM: monocyte derived macrophage
MIE: molecular initiating event
MTT: 3-[4,5-dimethylthiazol-2-yl]-2,5 diphenyl tetrazolium bromide
MWCNT: multiwalled carbon nanotube
OECD: Organisation for Economic Co-operation and Development
qRT-PCR: quantitative reverse transcription polymerase chain reaction
RNA: ribonucleic acid
SEM: scanning electron microscopy
TEM: transmission electron microscopy
TG: test guidelines
WST: water-soluble tetrazolium salts
